# Multi-task feature selection in microarray data by binary integer programming

**DOI:** 10.1186/1753-6561-7-S7-S5

**Published:** 2013-12-20

**Authors:** Liang Lan, Slobodan Vucetic

**Affiliations:** 1 Department of Computer and Information Science, Temple University, Philadelphia, PA, USA

## Abstract

A major challenge in microarray classification is that the number of features is typically orders of magnitude larger than the number of examples. In this paper, we propose a novel feature filter algorithm to select the feature subset with maximal discriminative power and minimal redundancy by solving a quadratic objective function with binary integer constraints. To improve the computational efficiency, the binary integer constraints are relaxed and a low-rank approximation to the quadratic term is applied. The proposed feature selection algorithm was extended to solve multi-task microarray classification problems. We compared the single-task version of the proposed feature selection algorithm with 9 existing feature selection methods on 4 benchmark microarray data sets. The empirical results show that the proposed method achieved the most accurate predictions overall. We also evaluated the multi-task version of the proposed algorithm on 8 multi-task microarray datasets. The multi-task feature selection algorithm resulted in significantly higher accuracy than when using the single-task feature selection methods.

## Background

Microarray technology has the ability to simultaneously measure expression levels of thousands of genes for a given biological sample, which is classified into one of the several categories (e.g., cancer vs. control tissues). Each sample is represented by a feature vector of gene expressions obtained from a microarray. Using a set of microarray samples with known class labels, the goal is to learn a classifier able to classify a new tissue sample based on its microarray measurements. A typical microarray classification data set contains a limited number of labeled examples, ranging from only a few to several hundred. Building a predictive model from such small-sample high-dimensional data is a challenging problem that has received a significant attention in machine learning and bioinformatics communities. To reduce the risk of over-fitting, a typical strategy is to select a small number of features (i.e., genes) before learning a classification model. As such, feature selection [1,2] becomes an essential technique in microarray classification.

There are several reasons for feature selection in microarray data, in addition to improving the classifier's generalization ability. First, the selected genes might be of interest to domain scientists interested in identifying disease biomarkers. Second, building a classifier from a small number of features could result in an easily interpretable model that could give important clues to biologists. Depending on how the feature selection process is combined with model learning process, feature selection techniques can be organized into three categories. (1) Filter methods [3] are independent of the learning algorithm. (2) Wrapper methods [4] are coupled with the learning algorithm using heuristics such as forward selection and backward elimination. (3) Embedded methods [5,6] integrate feature selection as a part of the classifier training. Both the wrapper and the embedded methods effectively introduce hyper-parameters that require computationally costly nested cross-validation and increase likelihood of over-fitting. Feature filter methods are very popular because they are typically conceptually simple, computationally efficient, and robust to over-fitting. These properties also explain why the filter methods are more widely used than the other two approaches in microarray data classification.

Traditional filter methods rank the features based on their correlation with the class label and then select the top ranked features. The correlation can be measured by statistic tests (e.g., *t*-test) or by information-theoretic criteria such as mutual information. The filter methods easily scale up to high dimensional data and can be used in conjunction with any supervised learning algorithm. However, because the traditional filter methods access each feature independently, highly correlated features tend to have similar rankings and tend to be selected jointly. Using redundant features could result in low classification accuracy. As a result, one common improvement for filter methods is to reduce redundancy between selected features. For example, minimal-redundancy-maximal-relevance (mRMR) proposed by [7] selects the feature set with both maximal relevance to the target class and minimal redundancy among the selected feature set. Because of the high computational cost of considering all possible feature sets, the mRMR algorithm selects features greedily, minimizing their redundancy with features chosen in previous steps and maximizing their relevance to the target class.

A common critique of popular feature selection filters is that they are typically based on relatively simple heuristics. To address this concern, recent research resulted in more principled formulation of feature filters. For example, algorithms proposed in [8] and [9] attempt to select the feature subset with maximal relevance and minimal redundancy by solving a constrained quadratic optimization problem (QP). The objective used by [8] is a combination of a quadratic term and a linear term. The redundancy between feature pairs is measured by the quadratic term and the relevance between features and class label is measured by the linear term. The features are ranked based on a weight vector obtained by solving a QP problem. The main limitation of this method is that the relevance between a feature and the class label is measured by either Pearson correlation or mutual information. However, Pearson correlation assumes normal distribution of the measurements, which might not be appropriate to measure correlation between numerical features and binary target. The mutual information requires using discrete variables and is sensitive to discretization. The objective used by [9] contains only one quadratic term. This quadratic term consists of two parts: one measures feature relevance using mutual information between features and the class label, and another measures feature redundancy using mutual information between each feature pair. However, the square matrix in the proposed quadratic term is not positive semi-definite. Thus, the resulting optimization problem is not convex and could result in poor local optima.

In this paper, we propose a novel feature filter method to find the feature subset which maximizes the inter-class separability and intra-class tightness, and minimizes the pairwise correlations between selected features. We formulate the problem as a quadratic programming with binary integer constraints. For high dimensional microarray data, to solve the proposed quadratic programming problem with binary integer constraints requires high time and space cost. Therefore, we relax binary integer constraints and apply the low rank approximation to the quadratic term in the objective function. The resulting objective function can be efficiently solved to obtain a small subset of features with maximal relevance and minimal redundancy.

In many real-life microarray classification problems, the size of the given microarray dataset is particularly small (e.g., we might have less than 10 labeled high-dimensional examples). In this case, even the most carefully designed feature selection algorithms are bound to underperform. Probably the only remedy is to borrow strength from external microarray datasets. Recent research [10,11] illustrates that multi-task feature selection algorithms can improve the classification accuracy. The multi-task feature selection algorithms select the informative features jointly across many different microarray classification data sets. Following this observation, we extend our feature selection algorithm to the multi-task microarray classification setup.

The contributions of this paper can be summarized as follows.(1) We propose a novel gene filter method which can obtain a feature subset with maximal discriminative power and minimal redundancy; (2) The globally optimal solution can be found efficiently by relaxing the integer constraints and using a low-rank approximation technique; (3) We extend our feature selection method to multi-task classification setting; (4) The experimental results show our algorithms achieve higher accuracy than the existing filter feature selection methods, both in single-task learning and multi-task settings.

## Results and discussion

We compared our proposed feature algorithm with 9 representative feature selection filters. The first 6 are standard feature selection filters: Pearson Correlation (PC), ChiSquare [3], GINI, Infogain, Kruskal-Wallis test and Relief [12]. They rank the features based on different criteria that measure correlation between each feature and class label. The remaining 3 are the state-of-the-art feature selection methods which are able to remove redundant features: mRMR [7], QPFS [8] and SASMIF [9]. The feature similarity for both QPFS and our algorithm was measured by Pearson correlation. For fair comparison, for the SASMIF method we used top *m *ranked features. To balance the effect of feature relevance and feature redundancy, the parameter λ in (9) was set to $\frac{m^{2}M\sum_{i}C_{i}}{\sum_{i,j}Q_{ij}}$. The low-rank parameter *k *was set to 0.1 · *M*, as suggested in [13]. Our algorithm is denoted as ST-BIP for single task version and MT-BIP for multi-task version.

Given the selected features, we used LIBLINEAR [14] to train the linear SVM model. The linear SVM model was chosen because previous studies [5] showed SVM classifier could be very accurate on microarray data. The regularization parameter *C *of LIBLINEAR was chosen among {10^-3^, 10^-4^, …, 10^3^}. For the experiments in the single-task scenario, we used the nested 5 cross validation to select the optimal regularization parameter. For experiments in multi-task learning scenario, it was too time consuming to use the nested cross-validation to select the regularization parameter. Thus, we simply fixed the regularization parameter to 1 in the multi-task experiments.

### Single task feature selection

In this section, we evaluate our proposed feature selection algorithm for single-task learning using four benchmark microarray gene expression cancer datasets: (1) Colon dataset [15] containing 62 samples, 40 tumor and 22 normal samples; (2) Lung dataset [16] containing 86 samples coming from 24 patients that died and 62 that survived; (3) Diffuse B-cell Lymphoma (DLBCL) dataset [17] containing 77 samples, 58 coming from DLBCL patients and 19 from Bcell lymphoma patients. (4) Myeloma dataset [18] containing 173 samples, 137 coming from patients with bone lytic lesions and 36 from control patients. We summarize the characteristics of these datasets in Table 1.

**Table 1:** Summary of the Microarray Datasets

	Colon	Lung	DLBCL	Myeloma
# Samples	60(40/22)	86(24/62)	77(58/19)	173(137/36)
# Genes	2000	5469	5469	12558

For each microarray dataset, we randomly selected 20 positive and 20 negative examples (except for choosing 15 positive and 15 negative in DLBCL dataset) as the training set and the rest as the test set. Due to the class imbalance in test sets, we used AUC, the area under the Receiver Operating Characteristic (ROC) curve, to evaluate the performance. The average AUC based on 10 repetitions of experiments on different random splits to training and test set are reported in Table 2. We Compared the AUC accuracy of different feature selection algorithms for *m *= 20, 50, 100, 200, 1000. For each dataset, the best AUC score among all methods was emphasized in bold. As shown in Table 2, our proposed method achieved the highest accuracy on Colon and DLBCL datasets. On the Myeloma dataset, it had the highest accuracy when *m *= 100 and 1000 and had the second highest accuracy when *m *= 20, 50 and 200. On the Lung dataset, our algorithm was ranked in the upper half of the competing algorithms. The last column in Table 2 shows the average AUC score across four different datasets. Our method achieved the highest average AUC scores. The next two successful feature selection algorithms are Relief and QSFS. The mRMR had somewhat lower accuracy, comparable to simple filters such as PC, ChiSquare, GINI and InfoGain. SASMIF was considerably less accurate, while KW was the least successful.

**Table 2:** Average AUC of 10 different feature selection algorithms on 4 different microarray datasets

		Colon	Lung	DLBCL	Myeloma	Average
m = 20	PC	.775 ± .159	.657 ± .184	.945 ± .051	.689 ± .094	.767
	ChiSquare	.763 ± .189	.573 ± .146	.945 ± .043	.639 ± .121	.730
	GINI	.760 ± .217	.590 ± .170	.948 ± .054	.653 ± .096	.738
	InfoGain	.758 ± .197	.546 ± .160	.948 ± .054	.639 ± .111	.723
	KW	.735 ± .145	.548 ± .165	.858 ± .099	.582 ± .112	.681
	Relief	.775 ± .149	**.685 ± .195**	.949 ± .043	.671 ± .104	**.770**
	mRMR	.785 ± .163	.556 ± .164	.938 ± .074	.649 ± .126	.732
	SASMIF	.710 ± .168	.560 ± .145	.931 ± .052	.612 ± .076	.703
	QSFS	.793 ± .129	.579 ± .186	.942 ± .043	**.737 ± .062**	.763
	ST-BIP	**.815 ± .153**	.612 ± .108	**.953 ± .054**	.701 ± .048	**.770**

m = 50	PC	.763 ± .170	.648 ± .184	.958 ± .025	.709 ± .071	.770
	ChiSquare	.740 ± .189	.600 ± .173	.965 ± .035	.676 ± .076	.745
	GINI	.742 ± .183	.586 ± .167	.966 ± .034	.666 ± .096	.740
	InfoGain	.755 ± .179	.595 ± .170	.963 ± .026	.682 ± .085	.749
	KW	.755 ± .187	.574 ± .163	.858 ± .128	.606 ± .072	.698
	Relief	.785 ± .145	**.661**±.**194**	.966 ± .027	.677 ± .082	.772
	mRMR	.748 ± .182	.651 ± .219	.948 ± .067	.695 ± .093	.761
	SASMIF	.663 ± .206	.563 ± .130	.943 ± .043	.636 ± .004	.701
	QSFS	.695 ± .208	.608 ± .054	.961 ± .031	**.714**±**.080**	.745
	ST-BIP	**.828 **± **.082**	.600 ± .124	**.969 **± **.034**	.710 ± .110	.**777**

m = 100	PC	.753 ± .176	.607 ± .122	.963 ± .025	.708 ± .062	.758
	ChiSquare	.745 ± .184	.631 ± .164	.966 ± .024	.688 ± .063	.758
	GINI	.748 ± .186	.594 ± .202	.965 ± .026	.698 ± .079	.751
	InfoGain	.750 ± .180	.631 ± .164	.967 ± .022	.690 ± .062	.760
	KW	.727 ± .188	.570 ± .206	.879 ± .113	.624 ± .071	.700
	Relief	.773 ± .177	.631 ± .176	.958 ± .042	.708 ± .066	.768
	mRMR	.758 ± .169	.608 ± .169	.966 ± .035	.690 ± .075	.756
	SASMIF	.785 ± .131	.611 ± .213	.950 ± .035	.647 ± .072	.748
	QSFS	.777 ± .173	**.636**±**.113**	.965 ± .025	.710 ± .073	.772
	ST-BIP	**.833 **± **.078**	.627 ± .180	**.975 **± **.033**	**.735 **± **.086**	**.793**

m = 200	PC	.760 ± .164	.632 ± .120	.973 ± .018	.704 ± .059	.767
	ChiSquare	.750 ± .165	.611 ± .198	.973 ± .030	.673 ± .072	.752
	GINI	.753 ± .165	.617 ± .199	.974 ± .019	.690 ± .064	.759
	InfoGain	.755 ± .165	.611 ± .198	.977 ± .017	.673 ± .072	.754
	KW	.735 ± .219	.571 ± .199	.878 ± .145	.637 ± .036	.705
	Relief	.758 ± .162	.621 ± .157	.979 ± .025	**.721**±**.076**	.770
	mRMR	.755 ± .155	.585 ± .169	.974 ± .027	.668 ± .068	.746
	SASMIF	.820 ± .011	.590 ± .124	.954 ± .221	.644 ± .045	.752
	QSFS	.765 ± .171	**.664 **±**.187**	.974 ± .025	.687 ± .052	.773
	ST-BIP	**.833 **± **.080**	.634 ± .156	**.984 **± **.020**	.706 ± .106	**.789**

m = 1000	PC	.740 ± .172	.633 ± .193	.979 ± .018	.700 ± .049	.763
	ChiSquare	.743 ± .174	.606 ± .121	.974 ± .028	.676 ± .060	.750
	GINI	.735 ± .176	**.645 **±**.152**	.974 ± .027	.679 ± .056	.758
	InfoGain	.743 ± .174	.606 ± .121	.974 ± .028	.676 ± .060	.750
	KW	.722 ± .198	.568 ± .184	.941 ± .051	.652 ± .037	.721
	Relief	.728 ± .173	.623 ± .150	.980 ± .019	.698 ± .051	.757
	mRMR	.743 ± .174	.606 ± .121	.976 ± .025	.677 ± .060	.751
	SASMIF	.763 ± .149	.587 ± .176	.952 ± .038	.669 ± .054	.743
	QSFS	.745 ± .175	.624 ± .163	.980 ± .017	.690 ± .047	.760
	ST-BIP	**.828 **± **.063**	.625 ± .192	**.981 **± **.020**	**.722 **± **.078**	**.789**

### Multi-task feature selection

In this section, we evaluate our proposed feature selection algorithm for multi-task learning. We used 8 cancer related binary microarray classification datasets published in [19]. The data are summarized in Table 3. As shown in Table 3, the size of the 8 microarray datasets was very small. The single-task feature selection algorithms are not expected to perform well because there might be insufficient information even when simple feature selection filters are used. In contrast, our multi-task feature selection algorithm is expected to improve the accuracy by borrowing strength across multiple microarray datasets.

**Table 3:** Multi-Task Microarray Datasets(cancer:normal case)

Bladder 18(11/7)	Lung 27(20/7)	Prostate 23(14/9)	Breast 22(17/5)
**Renal** 24(11/13)	**Colon** 26(15/11)	**Pancreas** 21(11/10)	**Uterus** 17(11/6)

**Figure 1:**
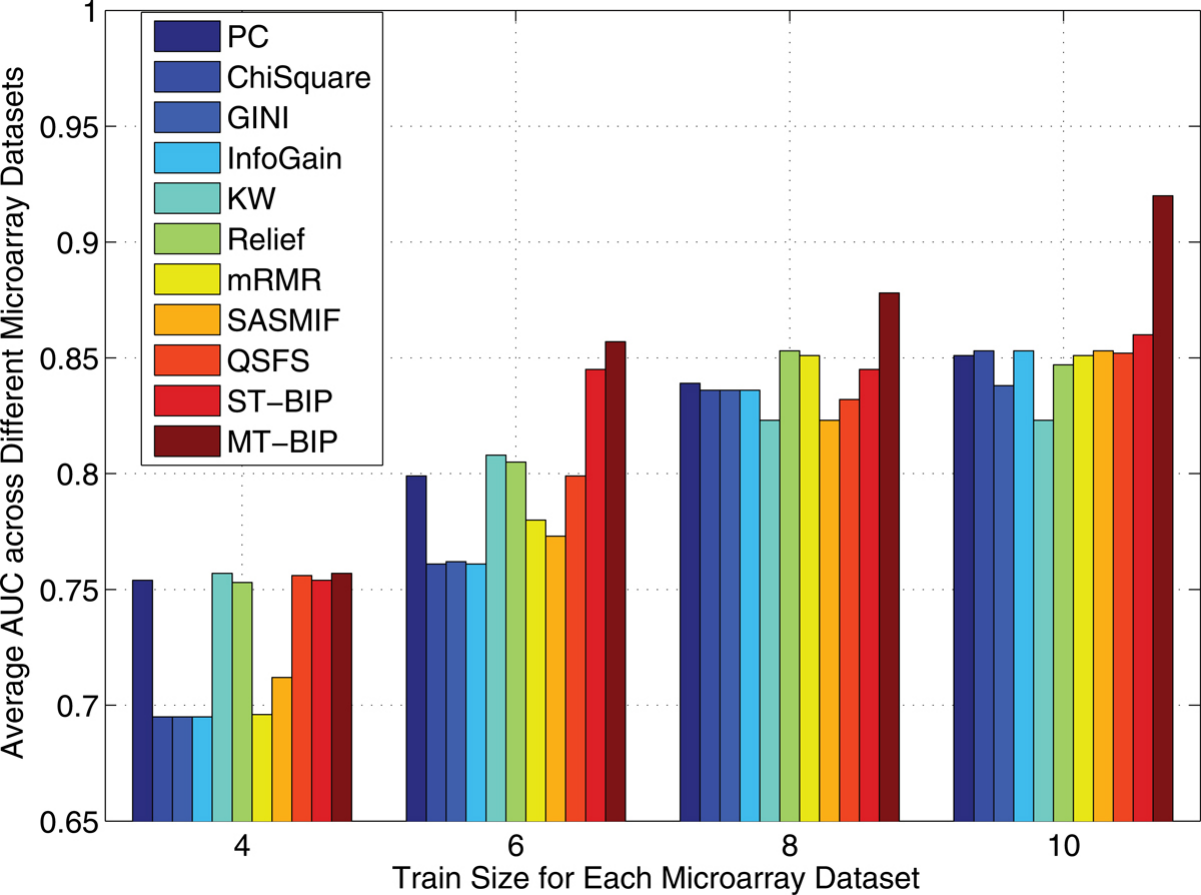
**Average AUC score of different feature selection algorithms across different train sizes**.

For each microarray data set, we randomly selected *N^+ ^*= 2, 3, 4, 5 positive and the same number of negative examples as the training data and used the rest as the test data. We show the results for *m *= 100 in this section. The average AUC across these 8 microarray datasets is shown in Figure 1. The results clearly show the multi-task version of our proposed algorithm was the most successful algorithm overall.

To gain a deeper understanding about the reason why the multi-task feature selection algorithm obtained better overall accuracy than single-task feature selection algorithms, we show the AUC score of each individual microarray dataset based on $N^{+}=3$ in Table 4. We can see that the single task version of our feature selection algorithm had the highest overall accuracy among other single-task benchmarks, a result consistent with Table 2. The multi-task version of our algorithm has higher AUC than its single task version on 4 datasets and its average AUC is about 1.5% higher. In 4 cases, (e.g. Colon, Lung, Pancreas, Renal datasets) we can also observe the negative transfer, where the accuracy drops. How to prevent negative transfer in multi-task feature selection would be another interest research topic for our future research.

**Table 4:** Average AUC of 11 different feature selection algorithms on 8 different microarray datasets

	Blad	Breast	Colon	Lung	**Panc**.	**Pros**.	Renal	Uterus	Ave
PC	.991	.696	.816	.703	.78	.603	.916	.883	.799
ChiSquare	.969	.625	.749	.669	.789	.636	.741	.908	.761
GINI	.969	.625	.749	.669	.789	.636	.743	.917	.762
InfoGain	.969	.625	.749	.669	.789	.636	.741	.908	.761
KW	.903	.621	.907	.750	.876	.626	.870	.913	.808
Relief	.991	.729	.795	.721	.796	.594	.929	.888	.805
mRMR	.969	.650	.765	.682	.830	.682	.786	.875	.780
SASMIF	.978	.739	.704	.671	.823	.650	.768	.854	.773
QSFS	.991	.693	.817	.700	.788	.600	.916	.883	.799
ST-BIP	.991	.679	**.921**	**.782**	**.882**	.612	**.966**	.910	.843
MT-BIP	**.997**	**.850**	.882	.754	.846	**.715**	.895	**.921**	**.858**

### Gene-annotation enrichment analysis for multi-task microarray datasets

The multi-task experimental results show that accuracies obtained by MT-BIP are better than other single task feature filters overall. So we would like to perform function annotation of the MP selected genes. In MT-BIP filter, only one selected gene list is obtained for all 8 different types of cancers. Given this gene list, the top 10 enriched GO terms were obtained using DAVID Bioinformatics Resources [20]. The top 10 enriched GO terms based on MT-BIP selected gene list is shown in Table 5. In this table, the hits means the number of genes that are found in the selected gene list associating with the specific GO term. The *p*-value was obtained by Fisher Exact test which is used to measure the gene-enrichment in annotation terms. After we got the enriched GO terms, we used the Comparative Toxicogenomics Database (CTD) [21] to check whether there is an association between the GO term and the cancer type. The last column in Table 5 shows the disease association for each GO term. The datasets are ordered as Bladder (B), Breast (B), Colon (C), Lung (L), Pancreas (P), Prostate (P), Renal (R) and Uterus (U). If a GO term is associated with the given type of cancer, we write down the cancer name. Otherwise, we put the symbol # in that position. We could see that the enriched GO terms based MT-BIP tends to associate many different types of cancer. As shown in Table 5, GO:0005856 (cytoskeleton), GO:0005886 (plasma membrane) and GO:0032403 (protein complex binding) were associated with 7 different cancers. GO:0030054 (cell junction) and GO:0015629 (actin cytoskeleton) are associated with 6 different cancers.

**Table 5:** Top 10 enriched GO terms based on 100 MT-BIP selected genes

Enriched GO Term	Hits	*p***-value**	Disease Association
GO:0005856 cytoskeleton	21	7.49e-6	#BCLPPRU
GO:0043232 intracellular non-	29	1.79e-5	########
GO:0043228 non-membrane-	29	1.79e-5	########
GO:0003779 actin binding	10	5.35e-5	#BCLPP##
GO:0008092 cytoskeletal	12	6.41e-5	########
GO:0030054 cell junction	11	2.31e-4	BBCLPP##
GO:0044459 plasma membrane part	24	2.53e-4	##C#P###
GO:0005886 plasma membrane	32	1.09e-3	#BCLPPRU
GO:0015629 actin cytoskeleton	7	2.22e-3	BBCLPP##
GO:0032403 protein complex	6	3.83e-3	#BCLPPRU

## Conclusion

We proposed a novel feature filter method to select a feature subset with discriminative power and minimal redundancy. The proposed feature selection method is based on quadratic optimization problem with binary integer constraints. It can be solved efficiently by relaxing the binary integer constrains and applying a low-rank approximation to the quadratic term in the objective. Furthermore, we extend our feature selection algorithm to multi-task classification problems. The empirical results on a number of microarray datasets show that in the single task scenario the proposed algorithm results in higher accuracy than the existing feature selection methods. The results also suggest that our multitask feature selection algorithm can further improve the microarray classification performance.

## Methodology

### Feature selection by binary integer programming

Let us denote the training dataset as *D *= (**x***_i_*, *y_i_*), *i *= 1, …, *N *, where **x***_i _*is an *M *dimensional feature vector for the *i*-th example and *y_i _*is its class label. *N *is the number of training examples. Our objective is to select a feature subset that is strongly predictive of class label and has low redundancy. We introduce a binary vector $w=\;{\left[w_{\text{1}},w_{\text{2}},\;.\;.\;.\;,w_{M}\right]}^{T}$ to indicate which features are selected:

(1) $$w_{j}=\left\{\begin{array}{cc} 1 & if\;feature\;j\;is\;selected\\ 0 & if\;feature\;j\;is\;not\;selected \end{array}\right)$$

So, the new feature vector for the *i*-th example after feature selection can be represented as **g***_i _*= **x***_i _*⊙ **w**, where the symbol ⊙ denotes the pairwise product. Therefore, *g_ij _*= *x_ij_*, for *w_j _*= 1 and *g_ij _*= 0 for *w_j _*= 0. Alternatively, **g***_i _*can be represented as **g***_i _*= ***W*x***_i_*, where *W *is a diagonal matrix and its diagonal is the vector **w**.

Intuitively, we would like the examples with the same class to be close (intra-class tightness) and the examples from different classes to be far away (inter-class separability) in the spaces defined by selected features. The Euclidean distance between two examples **x***_i _*and **x***_j _*in the new feature space can be calculated as

(2) $$d_{ij}\;=\;{||g_{i}-g_{j}||}^{2}=\;{||{\text{x}}_{i}⊙w-{\text{x}}_{j}⊙w||}^{2}=\;{||Wx_{i}-Wx_{j}||}^{2}$$

The inter-class separability of the data can be measured by a sum of the pairwise distances between examples with different class labels

(3) $$\underset{y_{i}\neq y_{j}}{\sum }{∥x_{i}⊙w-x_{j}⊙w∥}^{2}.$$

The intra-class tightness of the data can be measured by a sum of the pair-wise distances between examples with the same class label

(4) $$\underset{y_{i}=y_{j}}{\sum }{∥x_{i}⊙w-x_{j}⊙w∥}^{2}.$$

Therefore, the problem of selecting a feature subset to maximize the intra-class tightness and inter-class separability can be formulated as

(5) $$\underset{w}{\text{min}}\underset{y_{i}=y_{j}}{\sum }{∥x_{i}⊙w-x_{j}⊙w∥}^{2}-\underset{y_{i}\neq y_{j}}{\sum }{∥x_{i}⊙w-x_{j}⊙w∥}^{2}.$$

Objective (5) can be rewritten as

(6) $$\underset{w}{\text{min}}\overset{N}{\underset{i=1}{\sum }}\overset{N}{\underset{j=1}{\sum }}\;{∥x_{i}⊙w-x_{j}⊙w∥}^{2}A_{ij},$$

where matrix A is defined as:

(7) $$A_{ij}=\left\{\begin{array}{cc} 1 & if\;y_{i}=y_{j}\\ -1 & if\;y_{i}\neq y_{j} \end{array}\right)$$

In addition to the objective (5) or (6), in order to improve the diversity of selected features, we would like to select a feature subset with minimal redundancy. A feature is defined to be redundant if there is another feature highly correlated with it. Let us denote *Q *as a symmetric positive semidefinite matrix with size *M *× *M*, whose element *Q_ij _*represents the similarity between feature *i *and feature *j*. Since the measurements of each feature across different samples are normal distributed, it is reasonable to use Pearson Correlation to measure the similarity between two features here. Also, the similarity matrix *Q *is positive semi-definite when Pearson Correlation is used. Then, we define a redundancy among the selected set of features represented by vector **w **as their average pair-wise similarity **w^*T*^***Q***w***/m*^2^, where *m *is the number of selected features. Our objective is to minimize the redundancy defined in such way.

The first contribution of this paper is to formulate the feature selection task as a new quadratic programming problem subject to binary integer and linear constraints as follows,

(8) $$\begin{array}{c} \underset{w}{\text{min}}\overset{N}{\underset{i=1}{\sum }}\overset{N}{\underset{j=1}{\sum }}\;{||x_{i}⊙w-x_{j}⊙w||}^{2}A_{ij}+\lambda \frac{1}{m^{2}}w^{T}Qw\\ \;\;s.t.\;w_{i}\in \left\{0,1\right\}\forall i\\ \;\;\;\;\;\;\;\overset{M}{\underset{i=1}{\sum }}w_{i}=m. \end{array}$$

The first term in (8), which is a linear term as shown in the following **Proposition 1**, tries to maximize the interclass separability and intra-class tightness of the data. It describes the discriminative power of the selected feature subset. The second quadratic term is the average pair-wise similarity score between the selected features, which results in reduction of feature redundancy. Parameter λ is introduced to control the tradeoff between feature relevance and feature redundancy. Since *Q *is a positive semi-definite matrix, the proposed objective function is convex. The first constraint ensures that the resulting vector **w **is binary, while the second constraint ensures that exactly *m *features are selected. The following proposition establishes that the first term in the objective (8) is linear.

**Proposition 1**. *The first term of the objective function (8) can be written as a linear term **c**^T ^**w**, where **c **is vector of size M with elements c_i _*= (*X^T^LX*)*_ii_, L is the Laplacian matrix of A, defined as L *= *D *− *A. D is a diagonal degree matrix such that $D_{ii}=\sum_{j}A_{ij}$. The X is the N *× *M feature matrix. Each row in X corresponds to one example*. (*X^T^LX*)*_ii _denotes the i-th element in the diagonal of the matrix X^T^LX*.

*Proof*. Let us denote *W *as a diagonal matrix where *W_ii _*= *w_i_*. Then,

$$\begin{array}{cc} \overset{N}{\underset{i=1}{\sum }}\overset{N}{\underset{j=1}{\sum }}\;{||{\text{x}}_{i}⊙w-x_{j}⊙w||}^{2}A_{ij} & =\overset{N}{\underset{i=1}{\sum }}\overset{N}{\underset{j=1}{\sum }}{||W{\text{x}}_{i}-W{\text{x}}_{j}||}^{2}A_{ij}\\ & =trace\left(W^{T}X^{T}LXW\right)\\ & =trace\left(X^{T}LXWW^{T}\right) \end{array}$$

because *w_i _*∈ {0, 1}, *WW^T ^*= *W*. Therefore, $trace\left(X^{T}LXWW^{T}\right)=\sum_{i=1}^{M}{\left(X^{T}LX\right)}_{ii}W_{ii}=c^{T}w$, where *c_i _*= (*X^T ^LX*)*_ii_*

Based on **Proposition 1**, objective (8) can be rewritten as the following constrained quadratic optimization problem,

(9) $$\begin{array}{c} \underset{w}{\text{min}}c^{T}w+\lambda \frac{1}{m^{2}}w^{T}Qw\\ s.t.\;w_{i}\in \left\{0,1\right\}\forall i\\ \;\;\overset{M}{\underset{i=1}{\sum }}w_{i}=m. \end{array}$$

There are two practical obstacles in solving (9): (1) Binary constraint of variable **w**, and (2) feature similarity matrix *Q *is with size *M *× *M*, which implies high computational cost for high dimensional data. In the next two sections, we will first relax the binary constraint, and then we will apply a low-rank approximation to *Q*. The resulting constrained optimization problem can be solved very efficiently, with linear time with respect to the number of features *M*.

**Problem Relaxation**. Due to the binary constraint on the indictor vector **w**, it is difficult to solve (9) [9]. To resolve this, we first relax the binary constraint on **w **by allowing its elements *w_i _*to be within the range [0, *m*]. Then, (9) could be approximated by

(10) $$\begin{array}{c} \underset{w}{\text{min}}c^{T}w+\lambda \frac{1}{m^{2}}w^{T}Qw\\ s.t.\;w_{i}\geq 0\;\forall i\\ \;\;\overset{M}{\underset{i=1}{\sum }}w_{i}=m. \end{array}$$

Now, (10) becomes a standard Quadratic Programming (QP) problem. The optimal solution can be obtained by a general QP solver (e.g., MOSEK [22]).

**Low-rank Approximation**. The matrix *Q *in (10) is of size *M *× *M *. So, it results in high time and space cost if we work with high dimensional microarray data. Therefore, we would like to avoid the computational bottleneck by using low-rank approximation techniques.

The matrix *Q *in (10) is symmetric positive semidefinite. So, it can be decomposed as *Q *= *U*Λ*U^T^*, where *U *is a matrix of eigenvectors and Λ is a diagonal matrix with corresponding eigenvalues of *Q*. By setting $\alpha =\Lambda^{\frac{1}{2}}U^{T}w$, it follows that $w=U{\Lambda^{-}}^{\frac{1}{2}}\alpha$. Therefore, problem (10) can be rewritten as

(11) $$\begin{array}{c} \underset{\alpha }{\text{min}}{\text{c}}^{T}U\Lambda^{-\;\frac{1}{2}}\alpha +\lambda \frac{1}{m^{2}}\alpha^{T}\alpha \\ s.t.\;\;\;\;U\Lambda^{-\;\frac{1}{2}}\alpha \geq 0\\ \;\;1U\Lambda^{-\;\frac{1}{2}}\alpha =m. \end{array}$$

Typically, the rank of *Q *(let us denote it as *k*) is much smaller than *M*, k≪M. Therefore, we can replace the full eigenvector and eigenvalue matrices *U *and Λ by the top *k *eigenvectors and eigenvalues, resulting in an *M *× *k *matrix *U_k _*and a *k *× *k *diagonal matrix Λ*_k_*, without losing much information. Therefore, (11) is reformulated as

(12) $$\begin{array}{c} \underset{\alpha }{\text{min}}c^{T}U_{k}\Lambda_{k}^{-\;\;\frac{1}{2}}\alpha +\lambda \frac{1}{m^{2}}\alpha^{T}\alpha \\ s.t.\;U_{k}\Lambda_{k}^{-\frac{1}{2}}\alpha \geq 0\\ \;\;1U_{k}\Lambda_{k}^{-\;\frac{1}{2}}\alpha =m. \end{array}$$

Since *α *is a vector with length *k*, k≪M. the QP (11) is reduced to a new QP in a *k*-dimensional space with *M *+ 1 constraints. Once the solution *α *of (12) is obtained, the variable **w **in original space can be approximated by $w=U_{k}\Lambda_{k}^{-\frac{1}{2}}\alpha$.

Decomposing matrix *Q *requires *O*(*M*^3^) time, which is expensive in microarray data where *M *is large. Next we will show how to efficiently compute the top *k *eigenvectors and eigenvalues using Nystrom approximation technique [23]. Nystrom method approximates a *M *× *M *symmetric, positive semi-definite matrix *Q *by

(13) $$Q=E_{Mk}W_{kk}^{-1}E_{Mk}^{T}$$

where *E_Mk _*denotes the sub-matrix of *Q *created by selecting *k *of its columns, and *W_kk _*is a sub-matrix that corresponds to the intersection of the selected columns and rows. Sampling schemes in Nystrom method include random sampling [23], probabilistic sampling [24], and *k*-means based sampling [13]. We chose the *k*-means sampling in our experiments because [13] showed that it produces very good low-rank approximations at a relatively low cost. Given (13), we can easily obtain the low rank approximation of Q as

(14) $$Q=GG^{T}\;where\;G=E_{Mk}W_{kk}^{-\;\frac{1}{2}}.$$

As shown in the following Proposition 2, the top *k *eigenvectors and eigenvalues can be computed in *O*(*Mk*^2^) time using Nystrom method, which is much more efficient than doing eigen-decomposition of *Q*, which requires *O*(*M*^3^) time.

**Proposition 2**. *The top k eigenvectors U_k _and the corresponding eigenvectors *Λ*_k _of $Q=GG^{T}$ can be approximated as *Λ*_k _*= Λ*_G _and $U_{k}=GU_{G}\Lambda_{G}^{-\;\frac{1}{2}}$, where U_G _and *Λ*_G _are obtained by the eigen-decomposition of k *× *k matrix *$G^{T}G=U_{G}\Lambda_{G}U_{G}^{T}$

*Proof*. First, we observe that *U_k _*contains orthonormal columns.

$$\begin{array}{cc} U_{k}^{T}U_{k} & =\Lambda_{G}^{-\frac{1}{2}}U_{G}^{T}G^{T}GU_{G}\Lambda_{G}^{-\frac{1}{2}}\\ & =\Lambda_{G}^{-\frac{1}{2}}U_{G}^{T}U_{G}\Lambda_{G}U_{G}^{T}U_{G}\Lambda_{G}^{-\frac{1}{2}}=I. \end{array}$$

Next, we observe that

$$U_{k}\Lambda_{k}U_{k}^{T}=GU_{G}\Lambda_{G}^{-\frac{1}{2}}\Lambda_{G}\Lambda_{G}^{-\frac{1}{2}}U_{G}^{T}G^{T}=GG^{T}=Q$$

Our proposed feature selection algorithm is summarized in Algorithm 1. In the Algorithm 1, steps 1 to 5 require *O*(*Mk*^2 ^+ *k*^3^) time. QP in step 6 with *k *variables has a polynomial time complexity with respect to *k*. Step 7 requires *O*(*Mk*) time. Therefore, overall, the proposed feature selection algorithm is very efficient and it has linear time complexity with the number of features *M*.

**Algorithm 1 **Single-Task Binary Integer Program Feature Selection

**Input: **training data *X*, their labels *y*, regularized parameter *λ*, number of features *m*, low-rank parameter *k*.

**Output: ***m *selected features

1. Apply **Proposition 1 **to compute the vector **c**

2. Use *k*-means to select *k *landmark features for low-rank approximation of *Q*

3. Compute *E_Mk _*and *W_kk _*in (13)

4. Obtain low-rank approximation of *Q *by (14)

5. Apply **Proposition 2 **to compute the top *k *eigenvalue Λ*_k _*and eigenvector *U_k _*of *Q*

6. Obtain *α *by solving the lower dimensional QP problem(12).

7. Obtain **w **in original feature space as $w=U_{k}\Lambda^{-\;\frac{1}{2}}\alpha$

8. Rank the features according to the weight vector **w **and select the top *m *features

### Multi-task feature selection by binary integer programming

Multi-task learning algorithms have been shown to be able to achieve significantly higher accuracy than single task learning algorithms both empirically [11] and theoretically [25]. Motivated by these promising results, in this section, we extend our feature selection algorithm to the multi-task setting. The objective is to select features which are discriminative and non-redundant over multiple microarray datasets.

Let us suppose there are *K *different but similar classification tasks, and denote the training data of the *t*-th task as $D^{t}=\left\{\left({\text{x}}_{i}^{t},\;y_{i}^{t}\right),\;i=1,\;.\;.\;.\;,\;N^{t}\right\}$, where $N^{t}$ is the number of training examples of the *t*-th task. [10,11] proposed multi-task feature selection algorithms that use *ℓ*_1,2 _norm to regularize the linear model coefficients *β *across *K *different classification tasks. The *ℓ*_1,2 _norm regularizer over all *β*s across *K *classification tasks could be expressed as $\sum_{j=1}^{M}\left(\sum_{t=1}^{K}{||\beta_{t}^{j}||}_{2}\right)$, where $\beta_{t}^{j}$ is the coefficient of the *j*-th feature in the *t*-th task. Due to the *ℓ*_1 _norm on the *ℓ*_2 _norm of group of coefficients of each feature across *K *tasks, the *ℓ*_1,2 _norm regularizer selects the same feature subset across *K *tasks. However, the *ℓ*_1,2 _norm regularized problem is challenging to solve because the non-smoothness of the *ℓ*_1,2 _norm. In this section, we would like to show our proposed feature selection can be easily extended to multi-task learning version. The resulting objective optimization problem have the same form as objective (9), which can be solved efficiently as shown in previous section.

Let us denote **w***_t _*as the binary indicator defined in (1) to represent the selected feature subset of the *t*-th classification task. If we do not consider the relatedness between these *K *classification task, individual **w***_t _*could be obtained by applying **Algorithm 1 **to different classification tasks. Based on the conclusion given by [10,11], it would be beneficial to select the same feature subset across *K *related classification task. In our case, this is can be achieved by setting **w***_t _*= **w ∀ ***t*. Therefore, the same feature across *K *tasks, defined by vector **w**, can be obtained by solving the following optimization problem,

(15) $$\begin{array}{c} \underset{w}{\text{min}}\overset{K}{\underset{j=1}{\sum }}c_{j}^{T}w+\lambda \frac{1}{m^{2}}w^{T}\overset{K}{\underset{j=1}{\sum }}Q_{j}w\\ s.t.\;w_{i}\in \left\{0,1\right\}\forall i\\ \;\;\overset{M}{\underset{i=1}{\sum }}w_{i}=m. \end{array}$$

where **c***_j _*and *Q_j _*are the linear and quadratic terms of the QP corresponding to the *j*-th task. The details about how to compute the **c***_j _*and *Q_j _*are explained in the previous section. The technique of relaxing binary integer constraints and applying low-rank approximation to *Q *introduced in the previous section can be used to solve (15). The extended multi-task feature selection algorithm is also a feature filter. It can be used in conjunction with any supervised learning algorithm.

## Competing interests

The authors declare that they have no competing interests.

## Authors' contributions

LL and SV conceived the study and developed the algorithm. LL wrote the first draft of the manuscript. Both authors participated in the preparation of the manuscript and approved the final version.

## Declarations

Publication of this work was supported by the U.S. National Science Foundation Grant IIS-0546155.

This article has been published as part of *BMC Proceedings *Volume 7 Supplement 7, 2013: Proceedings of the Great Lakes Bioinformatics Conference 2013. The full contents of the supplement are available online at http://www.biomedcentral.com/bmcproc/supplements/7/S7.
